# An immune cell infiltration-related gene signature predicts prognosis for bladder cancer

**DOI:** 10.1038/s41598-021-96373-w

**Published:** 2021-08-17

**Authors:** Hualin Chen, Yang Pan, Xiaoxiang Jin, Gang Chen

**Affiliations:** Department of Urology, The First Affiliated Hospital of Chongqing Medical University, Chongqing, China

**Keywords:** Bladder, Cancer, Transcriptomics, Bioinformatics

## Abstract

To explore novel therapeutic targets, develop a gene signature and construct a prognostic nomogram of bladder cancer (BCa). Transcriptome data and clinical traits of BCa were downloaded from UCSC Xena database and Gene Expression Omnibus (GEO) database. We then used the method of Single sample Gene Set Enrichment analysis (ssGSEA) to calculate the infiltration abundances of 24 immune cells in eligible BCa samples. By weighted correlation network analysis (WGCNA), we identified turquoise module with strong and significant association with the infiltration abundance of immune cells which were associated with overall survival of BCa patients. Subsequently, we developed an immune cell infiltration-related gene signature based on the module genes (MGs) and immune-related genes (IRGs) from the Immunology Database and Analysis Portal (ImmPort). Then, we tested the prognostic power and performance of the signature in both discovery and external validation datasets. A nomogram integrated with signature and clinical features were ultimately constructed and tested. Five prognostic immune cell infiltration-related module genes (PIRMGs), namely *FPR1*, *CIITA*, *KLRC1*, *TNFRSF6B*, and *WFIKKN1*, were identified and used for gene signature development. And the signature showed independent and stable prognosis predictive power. Ultimately, a nomogram consisting of signature, age and tumor stage was constructed, and it showed good and stable predictive ability on prognosis. Our prognostic signature and nomogram provided prognostic indicators and potential immunotherapeutic targets for BCa. Further researches are needed to verify the clinical effectiveness of this nomogram and these biomarkers.

## Introduction

Bladder cancer (BCa) is one of the most common urinary malignancies and there are around ten thousand newly diagnosed BCa per year worldwide^1^. Approximately 3/4 BCa are non- muscle-invasive bladder cancer (NMIBC) at the initial time of being diagnosed^2^. Treatment strategies for NMIBC include transurethral resection of bladder tumor with or without intavesical chemotherapy or immunotherapy, while the primary treatment option for muscle-invasive bladder cancer (MIBC) is radical cystectomy combined with intavesical medicine therapy^3^. The patients of BCa can have a favorable prognosis if the cancerous cells are confined to sub-mucosal connective tissues. Disappointingly, the 5-year overall survival rate can be declined to lower than 15% from 80% once the lesions progress beyond the lining, into the surrounding bladder muscle and even other parts of the body^4^. Moreover, the conventional therapeutics cannot make a satisfactory outcome for patients of late-stage BCa^5^. In such conditions, development of new strategies and efficient therapeutics are urgently needed to improve the prognosis for patients with BCa.

Recently, the explosive growth of researches on immunotherapy made historic breakthroughs in various types of malignancies including prostate cancer, renal clear cell carcinoma, etc.^6,7^. Li et al. have confirmed the critical role of tumor microenvironment in the progression of BCa^8^. And significant heterogeneities have been found in genome, transcriptome and biological process among groups with different immune-infiltration abundances in BCa^9^. These works suggest the importance and necessity of exploration of the immune-related molecular mechanisms in BCa.

This study intended to quantify the immune cells’ infiltration abundances in BCa by single sample gene set enrichment analysis (ssGSEA), and found genes correlated with the prognostic immune cells by weighted correlation network analysis (WGCNA). Then, a signature based on hub genes was constructed to calculate the survival risk of patient of BCa. Additionally, we built a nomogram to predict the overall survival rate at 1-, 3- and 5-year follow up, based on the signature and clinicopathological parameters. The results of our study provided several potential immunotargets and evidences for further research in immunotherapy of BCa.

## Results

### Identification of shared prognostic immune cells

As listed in Table 1, five shared immune cells related to prognosis of BCa were identified, including cytotoxic cells, CD8+ T cells, T helper cells, T follicular helper cells (TFH), and Dendritic Cells (DC). The KM curves of the five common prognostic cells were presented in Supplementary Fig. 1a, which showed that high infiltration levels tumor samples had favorable prognosis.

**Table 1:** Identification of shared prognostic immune cells.

Immune cells	KM survival with log-rank test		Univariate Cox regression analysis		
	p-values	FDR	HR	p-values	FDR
**Cytotoxic_cells**	**< 0.001**	**0.011**	**0.117**	**0.002**	**0.031**
**CD8_T_cells**	**0.011**	**0.124**	**0.000**	**0.003**	**0.031**
**DC**	**0.029**	**0.124**	**0.189**	**0.025**	**0.084**
**T_helper_cells**	**0.020**	**0.124**	**0.001**	**0.005**	**0.043**
**TFH**	**0.026**	**0.124**	**0.009**	**0.012**	**0.064**
Neutrophils	0.031	0.124	4.106	0.120	0.233
T_cells	0.068	0.232	0.252	0.024	0.084
NK_CD56bright_cells	0.118	0.291	0.121	0.013	0.064
Tem	0.121	0.291	1.316	0.846	0.923
Th17_cells	0.103	0.291	0.293	0.061	0.160
Tgd	0.228	0.497	0.288	0.136	0.233
NK_cells	0.280	0.559	28.342	0.104	0.228
B_cells	0.484	0.613	0.097	0.163	0.252
Eosinophils	0.362	0.613	0.009	0.067	0.160
Macrophages	0.390	0.613	2.388	0.168	0.252
Mast_cells	0.436	0.613	3.444	0.131	0.233
NK_CD56dim_cells	0.349	0.613	1.005	0.995	0.995
Th2_cells	0.413	0.613	29.974	0.038	0.113
TReg	0.485	0.613	0.548	0.327	0.436
Th1_cells	0.522	0.626	1.584	0.639	0.731
iDC	0.605	0.691	1.013	0.992	0.995
Tcm	0.716	0.781	0.257	0.468	0.562
aDC	0.781	0.815	0.608	0.421	0.532
pDC	0.989	0.989	0.536	0.287	0.406

The first five immune cells in bold were considered as the share prognostic ones.

*KM* Kaplan–Meier, *HR* hazard ratio, *FDR* p-values adjusted in FDR method.

The results of significant relationship between clinicopathological factors and prognostic cells were demonstrated in Supplementary Fig. 1b. The infiltration abundance of T helper cells was significantly lower in samples of elder patients, high grade, late stage (stage III and IV) and MIBC subtype. TFH also showed lower infiltration level in samples of late stage. The infiltration abundance of CD8 T cells, however, was significantly higher in MIBC, which may be influenced by the basis of small sample size of NMIBC.

### Development of a co-expression network and identification of the key module

**Figure 1:**
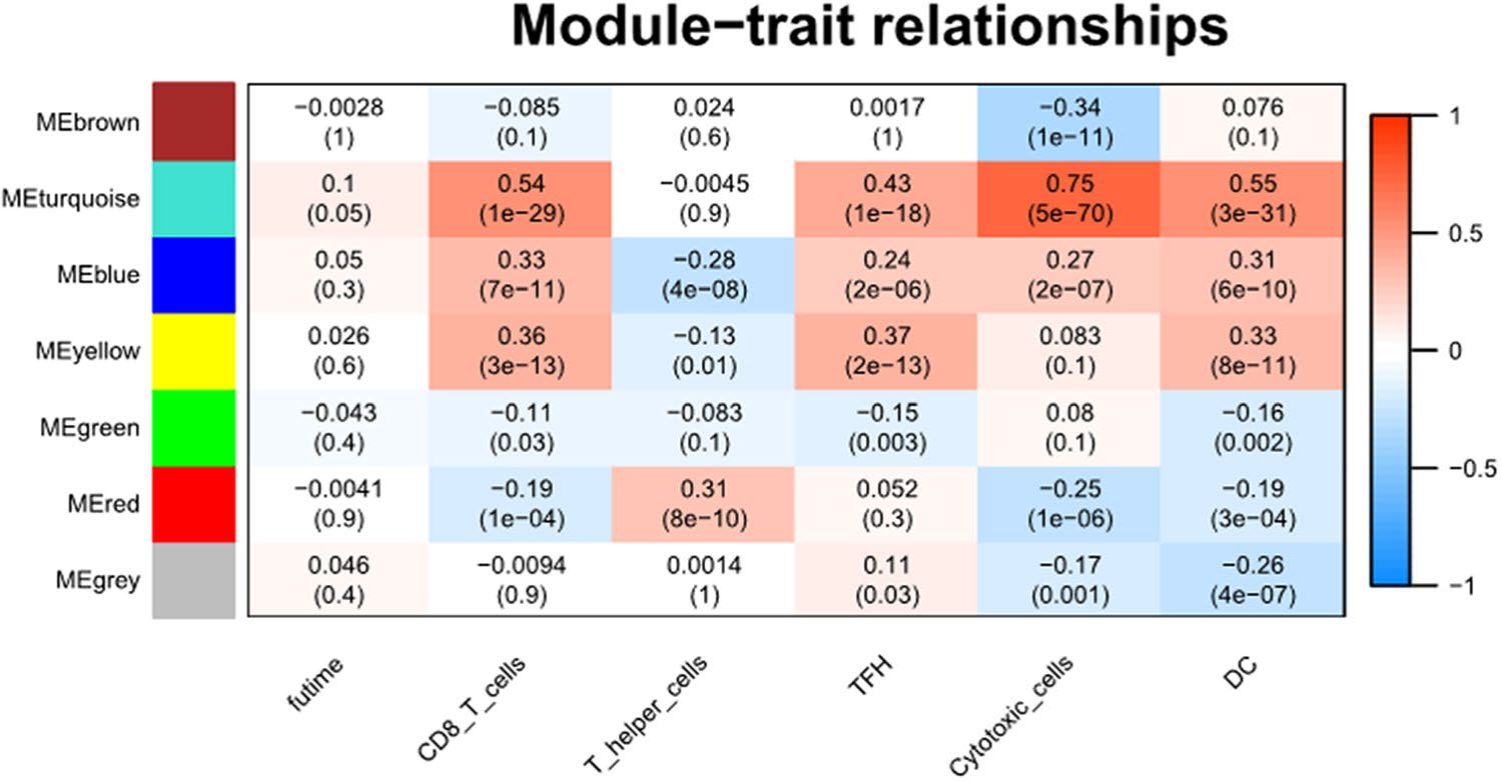
Identification of key module associated with the prognostic immune cells through WGNCA by module-trait heatmap. Each cell contains correlation and p value. Each column represents a trait. The module name is shown to the left side of each cell. Each module represents a cluster of densely interconnected genes in terms of co-expression.

Firstly, we constructed the sample dendrogram and trait heatmap with no outlier sample detected. Then, under the soft-thresholding of 3, the gene cluster dendrogram was produced with the high similarity of feature genes into the same module. Next, we created a module-trait heatmap to demonstrate the correlation between the infiltration levels of the five prognostic immune cells and different modules. As showed in Fig. 1, turquoise module showed the highest correlation coefficient with CD8 T cells (cor = 0.54), TFH (cor = 0.43), cytotoxic cells (cor = 0.75) and DC (cor = 0.55) simultaneously. Additionally, this module had marginally significant and weak correlation with the survival time compared to other modules. Taking together, this module was regarded as the key module and genes within the module were the most relevant to tumor prognosis. We then extracted all genes in this module for next studies.

### Identify IRMGs and perform enrichment analysis

**Figure 2:**
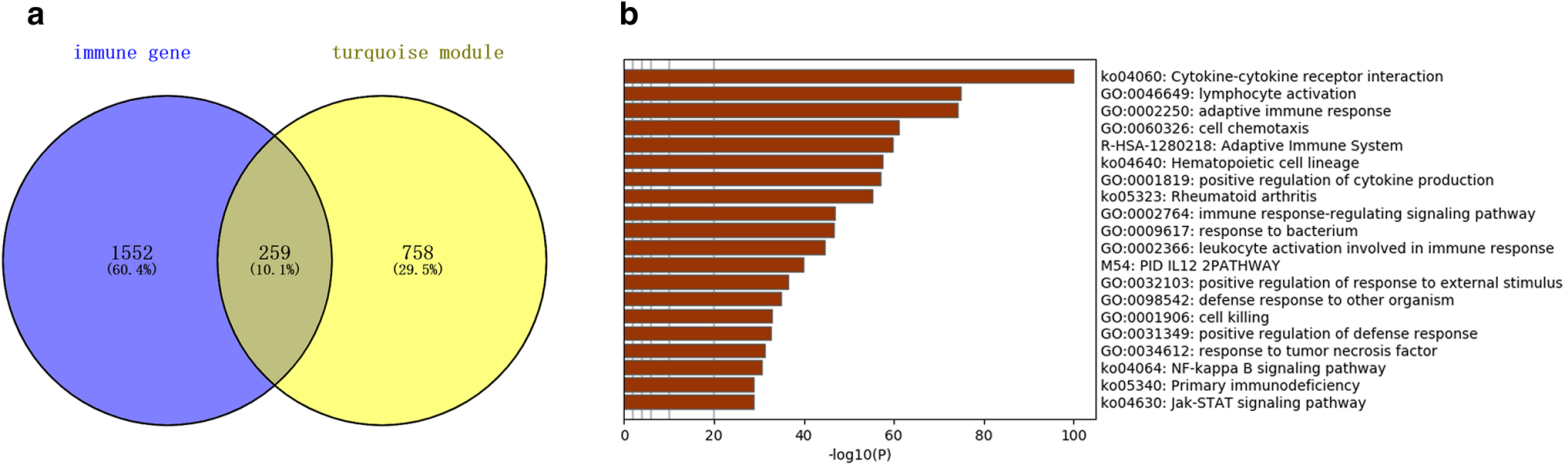
IRMGs identification and enrichment analysis. (**a**) 259 IRMGs (overlapping area) screened out by intersection of 2498 IRGs (circle at left) and 1017 MGs (circle at right). (**b**) The top 20 enriched clusters in Metascape.

As shown in Fig. 2a, a total of 259 shared genes of IRGs and MGs were identified and considered as immune-related module genes (IRMGs).

The top 20 enriched terms by the online tool Metascape were listed in Fig. 2b. The highest-levels of enriched clusters in the four categories were cytokine-cytokine receptor interaction in KEGG pathway, lymphocyte activation in biological process of GO, Adaptive Immune System in Reactome Gene Sets, and PID IL12 2PATHWAY (IL12-mediated signaling events) in Canonical pathways.

### PIRMGs identification, mutation analysis and TF regulatory network construction

**Figure 3:**
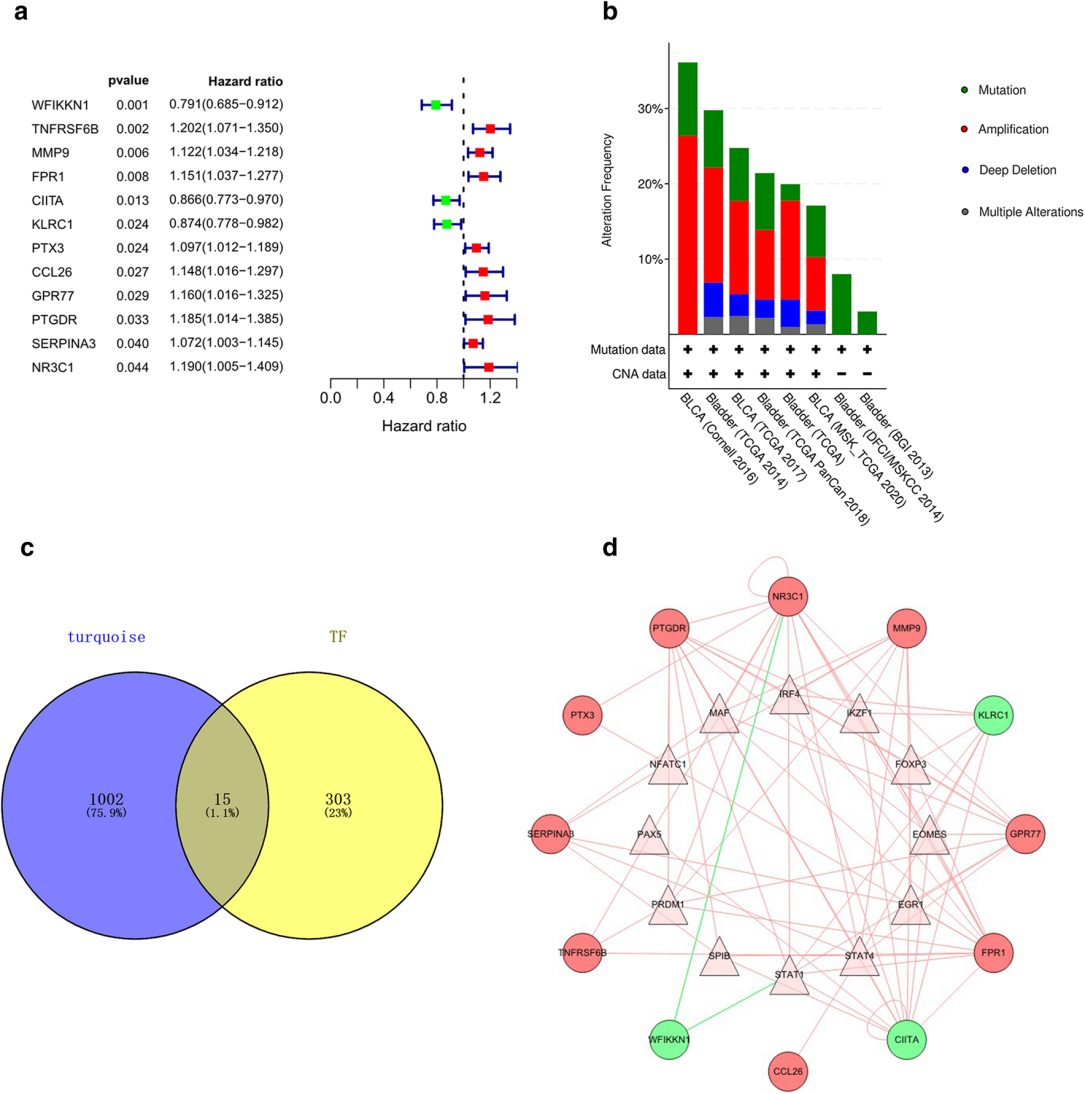
Regulatory network analysis of PIRMGs and module-related TFs. (**a**) A forest plot demonstrated the 12 IRMGs identified by univariate Cox regression analysis (p-value < 0.05). Right panel illustrated the hazard ratio of 12 IRMGs, in which red box indicated HR > 1 and light-blue indicated HR < 1. (**b**) Alteration analysis of PIRMGs by online database cBioPortal. The “+” and “−” symbols indicated that whether the alterations in DNA levels can be detected or not in corresponding dataset. (**c**) Identification of 15 module-related TFs (overlapping area) by intersection of 1017 MGs (circle at left) and 318 TFs (circle at right). (**d**) A regulatory network of the significant correlations of PIRMGs (circle) and module-related TFs (triangle). Lines indicated correlations. Red or light-blue indicated positive or negative correlation, respectively.

376 samples were randomly and equally divided into training and testing cohorts. We then performed univariate Cox regression analysis on the IRMGs in training cohort and identified 12 IRMGs related to the prognosis of BCa (Fig. 3a). Subsequently, to clarify the molecular characteristics of the PIRMGs, we used the online tool cBioportal to perform the gene alternations analysis and found that the most common types were amplification and mutation (Fig. 3b).

We then distinguished the TFs from the turquoise module and constructed a regulatory network based on the 12 PIRMGs and 15 module-related TFs (Fig. 3c,d).

### Develop a signature in the training cohort

The prognostic signature was developed with five PIRMGs, namely *FPR1*, *CIITA*, *KLRC1*, *TNFRSF6B*, and *WFIKKN1* (Table 2). The risk score calculator was: Risk score = 0.148502 × Exp.*FPR1* − 0.23798 × Exp. *CIITA* − 0.1929 × Exp. *KLRC1* + 0.190133 × Exp. *TNFRSF6B* − 0.24818 × Exp. *WFIKKN1*.

**Table 2:** Information of the five PIRMGs in the signature.

Gene symbol	Ensemble ID	Coef	HR	p-value	FDR
FPR1	ENSG00000171051	0.149	1.160	0.032	0.032
CIITA	ENSG00000179583	− 0.238	0.788	0.003	0.008
KLRC1	ENSG00000134545	− 0.193	0.825	0.016	0.020
TNFRSF6B	ENSG00000243509	0.19	1.209	0.006	0.010
WFIKKN1	ENSG00000127578	− 0.248	0.780	0.003	0.008

*Coef* regression coefficient calculated by multivariate Cox regression analysis, *HR* hazard ratio, *CI* confidence interval, *FDR* p-values adjusted in FDR method.

**Figure 4:**
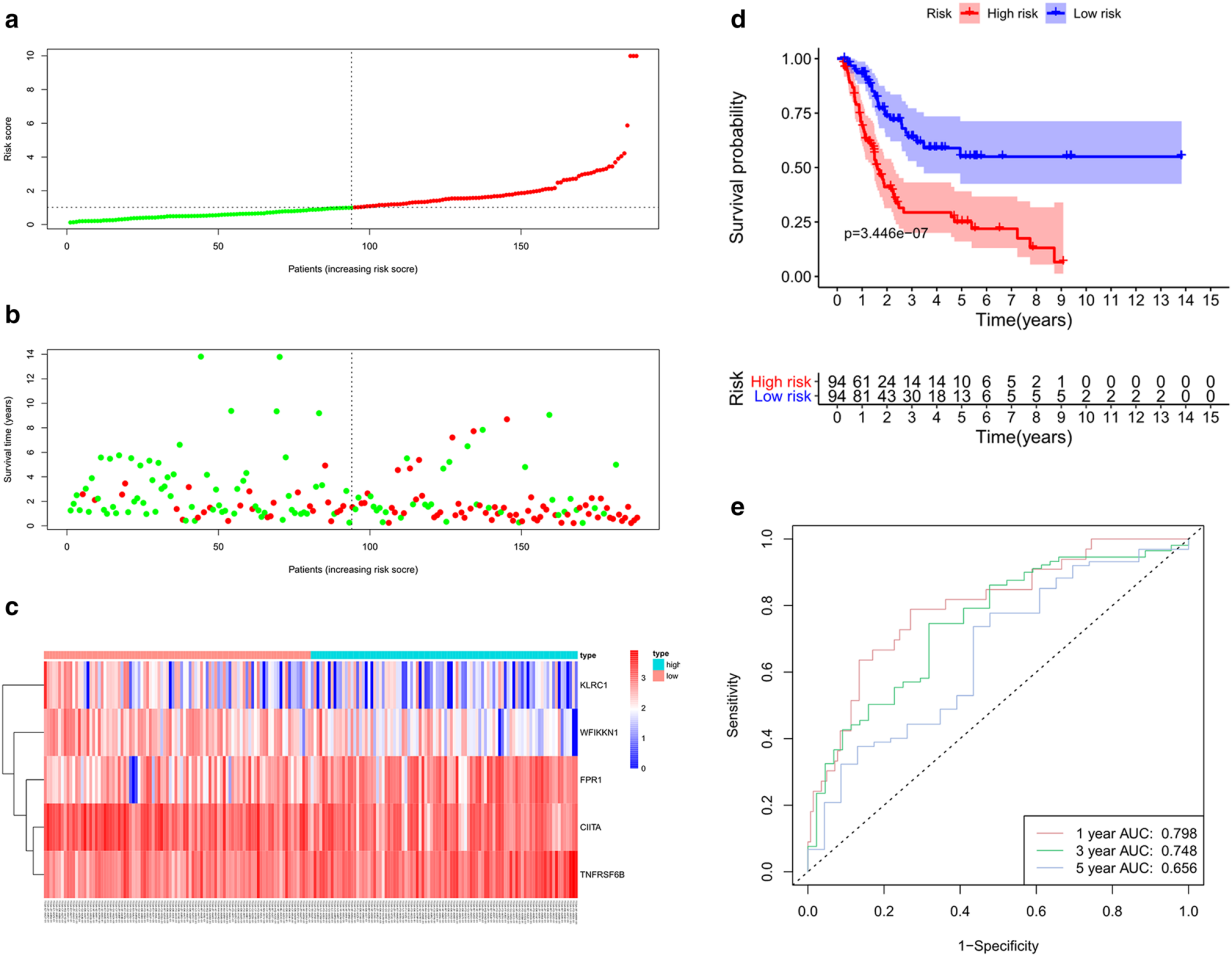
Evaluation of the prognosis prediction power of the five PIRMGs signature in the training group. (**a**) Distribution of risk score. (**b**) Survival status of BCa patients. (**c**) The expression profiles of the five PIRMGs. (**d**) KM survival analysis of the high- and low-risk groups. (**e**) Time-dependent ROC curve of the signature.

Next, 188 BCa samples in the training cohort were divided into high- and low-risk groups according to the median risk score of 1.018. Figure 4a–c demonstrated the risk score distribution, survival status and the five PIRMGs expression patterns between two groups. To assess the prognostic value of the signature, we performed KM survival analysis and found that patients in high-risk group had worse prognosis compared to those in low-risk group (Fig. 4d). We then produced time‐dependent ROC curves to estimate the performance of the signature, and found that area under curve (AUC) for 1‐, 3- and 5-year survival prediction was 0.798, 0.748 and 0.656, respectively (Fig. 4e).

### Signature validation in internal and external validation datasets

Based on the risk score calculator proposed in the training cohort, the risk score of each BCa sample of both internal and external validation datasets were obtained and these samples were further divided into high- and low-risk groups according to the median risk score in training group.

**Figure 5:**
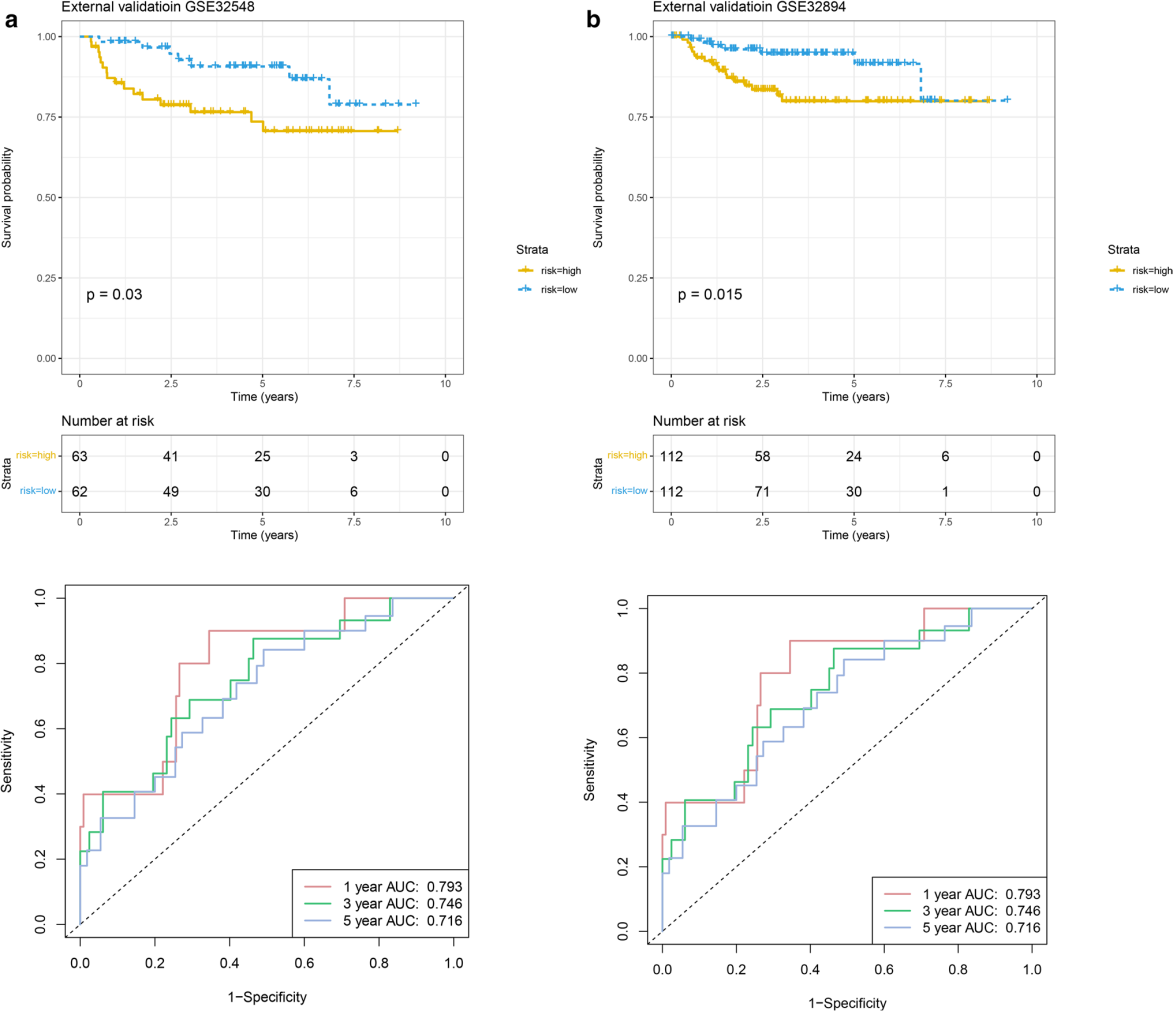
Evaluation of the prognosis prediction power of the five PIRMGs signature in (**a**) GSE32548 and (**b**) GSE32894 by KM survival analysis of the high- and low-risk groups and time-dependent ROC curves.

In internal validation dataset, the results were similar to those of the training cohort (Supplementary Fig. 2). Additionally, the results of the external validation datasets also suggested that BCa patients in high-risk group also suffered from worse prognosis than those in low-risk group (Fig. 5). Moreover, the signature presented stable performance in all cohorts.

In conclusion, the five PIRMGs signature can divided BCa patients into two risk-level groups with significant differences in overall prognosis.

### Independent analysis of the signature and clinicopathological characteristics

As shown in Table 3, the signature showed independent prognostic prediction ability in three cohorts of TCGA dataset, simultaneously. Besides, age and stage were considered as two independent clinicopathological predictors of BCa.

**Table 3:** Independence analysis results of the five PIRMGs signature in three cohorts.

Cohorts	Variable	Univariate			Multivariate		
		HR	p-value	FDR	HR	p-value	FDR
Training (n = 188)	Age	1.037	0.038	0.076	1.027	0.016	0.042
	Gender	1.409	0.608	0.644	1.325	0.243	0.437
	Grade	3.724	0.283	0.340	0.994	0.995	0.995
	Subtype	2.003	0.852	0.852	0.750	0.590	0.724
	Stage	2.150	0.014	0.033	1.975	< 0.001	< 0.001
	Risk score	1.462	< 0.001	< 0.001	1.314	< 0.001	< 0.001
Testing (n = 188)	Age	1.025	0.001	0.003	1.020	0.099	0.198
	Gender	0.871	0.133	0.218	0.824	0.475	0.660
	Grade	2.953	0.192	0.266	1.699	0.603	0.724
	Subtype	1.083	0.175	0.263	0.729	0.477	0.660
	Stage	1.447	< 0.001	< 0.001	1.400	0.032	0.072
	Risk score	1.306	< 0.001	< 0.001	1.293	0.001	0.002
Entire (n = 376)	Age	1.031	< 0.001	0.000	1.024	0.004	0.012
	Gender	1.144	0.438	0.493	1.057	0.755	0.799
	Grade	3.285	0.095	0.171	1.381	0.655	0.737
	Subtype	1.441	0.264	0.339	0.750	0.398	0.652
	Stage	1.773	< 0.001	< 0.001	1.633	< 0.001	< 0.001
	Risk score	1.400	< 0.001	< 0.001	1.326	< 0.001	< 0.001

*HR* hazard ratio, *Subtype* muscle-invasive BCa and non-muscle-invasive BCa, *FDR* p-values adjusted in FDR method.

**Figure 6:**
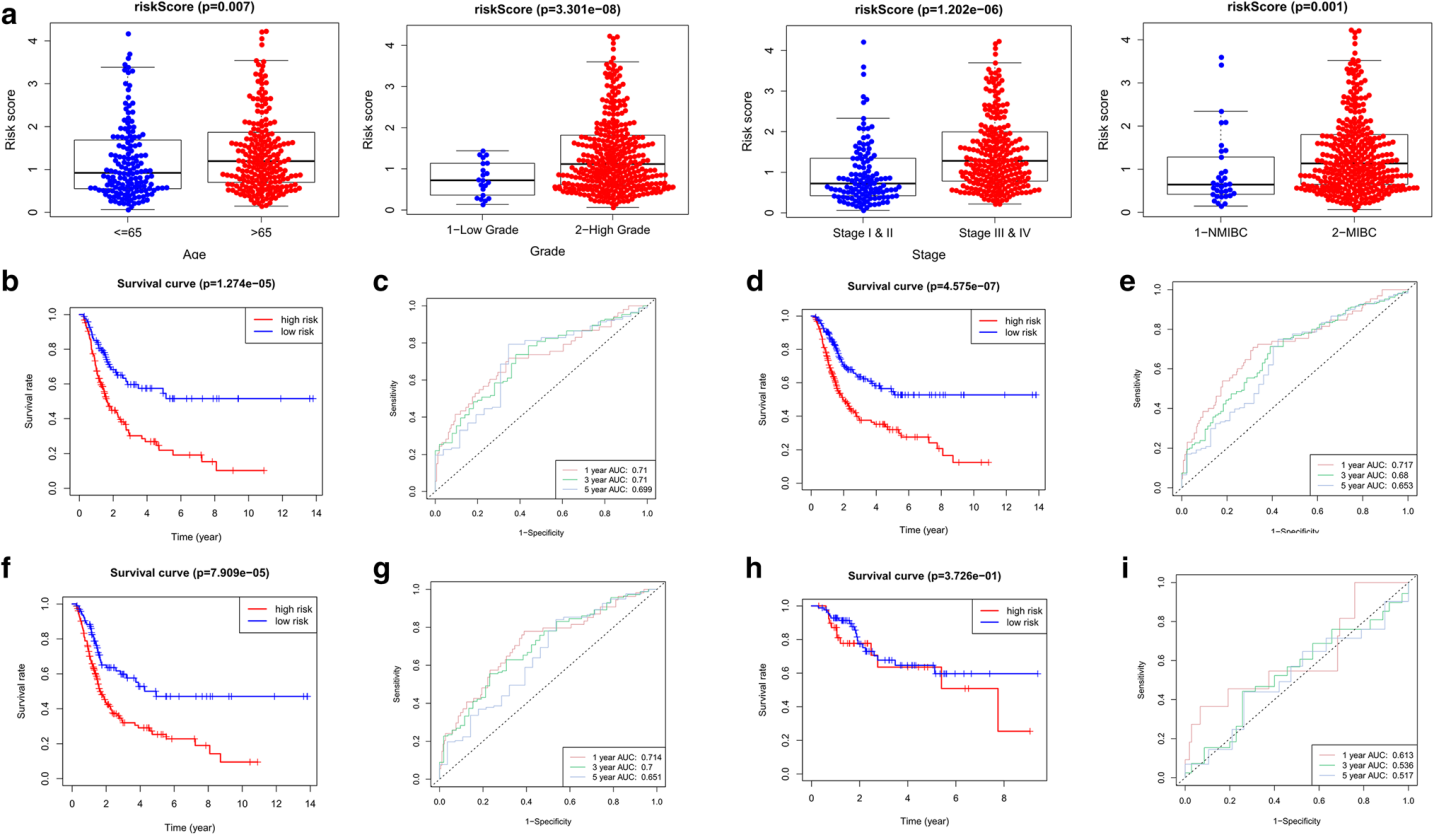
Relationship between risk score/signature and clinical characteristics. (**a**) Analysis of risk score in age, grade, stage and subtype. Results of stratified analysis: (**b**,**c**), age > 65; (**d**,**e**), age ≤ 65; (**f**,**g**) stage III and IV; (**h**,**i**) stage I and II.

The results of clinical features relationship analysis demonstrated that patients of high grade, late stage, MIBC subtype, and older age had significant higher risk scores (Fig. 6a).

Stratified analysis was further carried out between the signature and age and stage. And the results showed that the signature had significant prognostic value and reliability for BCa patients with the same age and late stage (Fig. 6b–i).

In short, these results suggested the relationship between signature and progression of BCa.

### Construction of a nomogram

**Figure 7:**
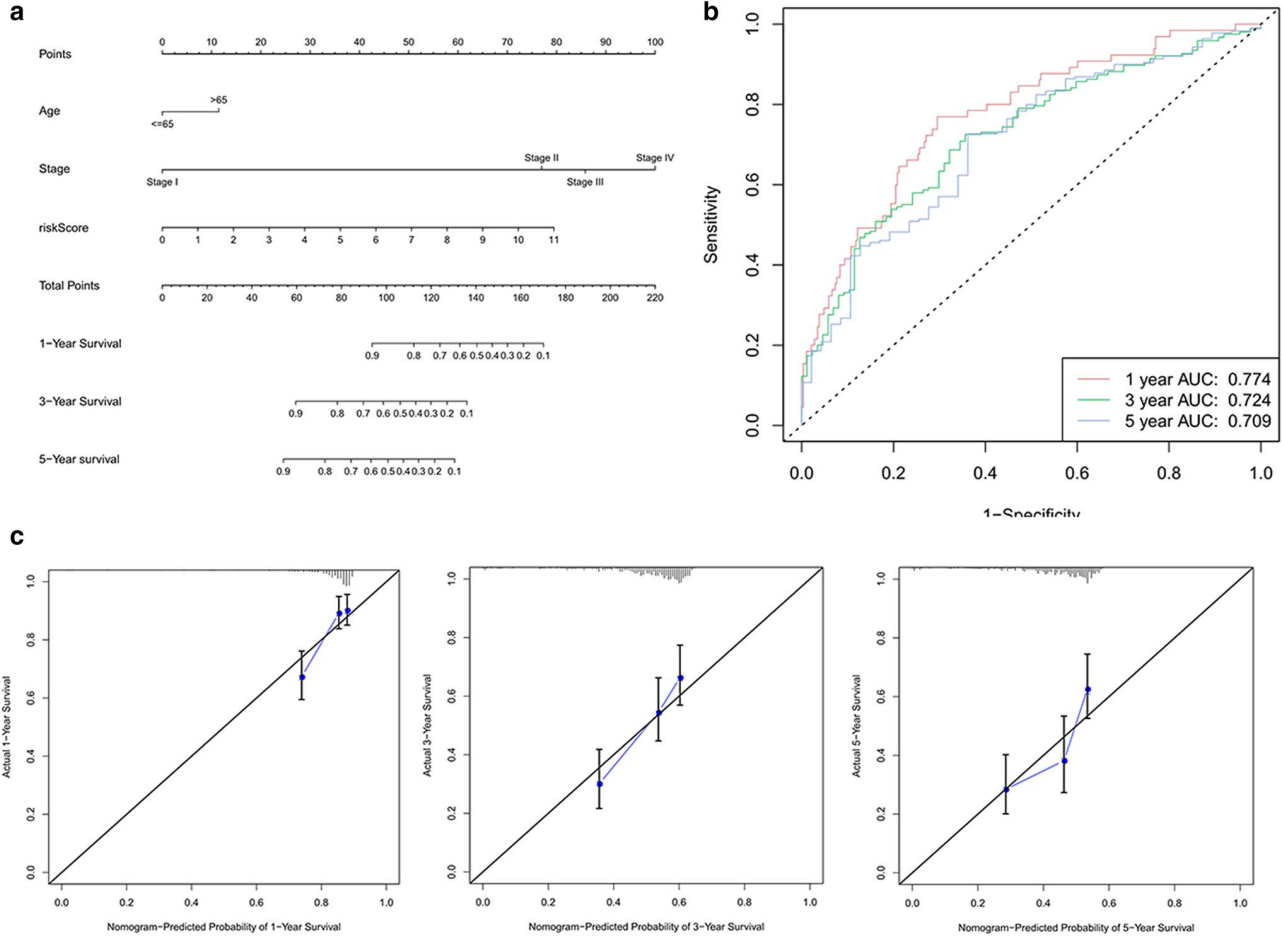
Building and evaluating the predictive power of the nomogram. (**a**) A nomogram constructed based on the five PIRMGs signature, age and stage. (**b**) Time-dependent ROC curves of the nomogram in the prediction of prognosis at 1-, 3-, and 5-year time points. (**c**) Calibration plots of the nomogram in 1-, 3-, and 5-year survival. The predicted and the actual probabilities of survival are plotted using blue and black solid lines, respectively.

Based on the five PIRMGs signature and age and stage, we constructed a nomogram to predict the 1-, 3-, and 5-year overall survival of BCa (Fig. 7a). Time-dependent ROC curves showed the adequate discrimination of the nomogram with an AUC of 0.774, 0.724, and 0.709 at 1-, 3-, and 5-year follow up (Fig. 7b). Additionally, we plotted the calibration plots to demonstrated the good predictive effect of this nomogram on overall survival (Fig. 7c).

In traditional clinical practices, some clinical variables such as age, tumor stage and grade were widely used in evaluating the survival probability of patients of BCa. Hence, to further test the clinical utility of our signature, we designed a full model composed of risk score and clinical variables, and a base model only integrating clinical variables. As illustrated in Supplementary Fig. 3, the full model demonstrated superiority to the based model in 1-, 3-, and 5-year time point. Moreover, we compared the AUC value of the nomogram to those of other clinical variables by ROC curves (Supplementary Fig. 4). In a word, the signature combined with traditional clinical variables has potential clinical utility.

## Discussion

In this study, we identified five PIRMGs (*FPR1*, *CIITA*, *KLRC1*, *TNFRSF6B*, and *WFIKKN1*) from the turquoise module which showed positive and significant correlations with several immune cells including CD8+ T cells. A prognostic signature was then constructed to divide patients of BCa into two distinct risk groups. Ultimately, a nomogram composing of signature, age and tumor stage was developed to calculate the total score of each BCa and return a quantitative survival probability. Two GEO datasets were obtained to validate the significant predictive power and good performance of the signature and nomogram, respectively. Although there have been several immune-related signatures proposed in literature, we firstly proposed a novel immune cell infiltration abundance-related prognostic signature of BCa through WGCNA.

Though correlation analysis between TFs of the turquoise module and PIRMGs, three key TFs (STAT4, IKZF1 and STAT1) were identified from the network. STAT4 and IKZF1 have been proved as essential factors in immune cell development and immune response^10^. STAT1 can affect the cellular survival and response to pathogens due to its critical roles in gene expression.

Cytotoxic cells are composed of CD8+ T cells, gamma delta T cells (Tγδ) and natural killer (NK) cells, participating in the innate immune system. The definite function of cytotoxic cells is eliminating intracellular pathogens and abnormal cells. Unlike CD8 T cells with strict major histocompatibility complex (MHC) restriction, both Tγδ and NK cells have the ability to recognize and kill infected cells in the absence of antibodies and MHC, and secret a large number of cytokines, allowing for a rapid immune response^11^. As a critical role in the adaptive immune system, T helper cells regulate the proliferation of B cells and participate in pathogen clearance, and autoimmunity through specific coordinate effector functions. There are two major subsets of T helper cells (Th1 and Th2 cells) once the proliferating T helper cells develop into effector T cells. Th1 cells mainly lead to an activated cell-mediated response, while Th2 cells favor a predominantly humoral response^12^. TFH, a subset CD4+ T cells, are found in B cell follicles and germinal centers. TFH play an important role in regulating the selection and survival of B cells that can differentiate into plasma cells and memory B cells. More importantly, TFH are believed to have the ability to decrease the repertoire of potentially autoimmune-causing mutated B cells within the germinal center. In general, the functions of, and mediation between, the cytokine, immune cells, and immune systems are critical for anti-tumor immunity, and profound understanding in these molecular interactions can promote the breakthroughs in tumor immunotherapies.

*WFIKKN1*, encodes large extracellular multidomain proteins, were mainly explored in cell growth and metabolism^13^, while, the biological behavior of *WFIKKN1* was poorly studied in tumors.

As reported in previous studies, the expression of peptide-loaded HLA class I molecule (HLA-E) in tumor cells can negatively mediate the anti-tumor activity of NK cell, by ligation of the NK inhibitory receptor CD94/*NKG2A* (*KLRC1*)^14^. This key molecular mechanism in tumor resistance to immune cells was further explored by Kamiya et al. via establishing *NKG2A*null and *NKG2A* + NK cells. And the results showed that *NKG2A* downregulation was associated with the higher cytotoxicity of NK cell in decreasing HLA-E-expressing tumor cells^15^. The consistent result was also reported in the study by Chen et al. who further found that *NKG2A* + CD8+ T cells form the predominant subset of *NKG2A*+ cells in lung cancer tissue but not NK cells and *NKG2A* blockade could promote anti-tumor immunity by reducing dysfunctional CD8+ T cells^16^. Surprisingly, these findings were contrary to the results of our study in which high expression of *NKG2A* was associated with favorable prognosis. Further studies are required to explore the role and molecular mechanism of *NKG2A* in BCa.

The critical role of *CIITA* in immune response to tumor cells was well established in literature. Mortara et al. reported that MHC class II expression in breast cancer was dependent on *CIITA*. And they found that *CIITA*-induced MHC class II expression on tumor cells had the ability of triggering an adaptive and protective immunity by presenting tumor antigen to Th cells, antitumor polarization, and establishment of antitumor immune memory^17^. Accolla et al. proposed an innovative method for construction of optimal anti-tumor vaccine, based on the biological functions of *CIITA*-induced MHC class II. The reason why traditional tumor-specific MHC-I-bound peptides had limited anti-tumor efficacy, as they analyzed, was that Th cell was inadequate triggered to maintain the proliferation of all the immune effector cells. Considering this drawback, they conducted a in vivo in mice assay and found that *CIITA*-driven MHC class II expression in tumor cells revealed strong inhabitation of tumor growth^18^. The protective role of *CIITA* in tumor was also proved by Lee et al.^19^. Based on these findings, novel anti-tumor vaccination protocols will be established and translated in clinics to provide a favorable prognosis for patients with tumors.

*FPR1* functions as a key part of the innate immune system mainly expressed in the phagocytic and blood leukocyte cells, and mediates the response to pathogens invasion. Jiang et al. reported that overexpression of *FPR1* was associated with drug-resistant BCa and may deteriorate the overall condition of drug-resistant BCa^20^. The biological behaviors of *FPR1* were also explored in ovarian cancer^21^, cervical cancer^22^, and lung cancer^23^ as a risk factor related to poor prognosis, advanced stage and metastasis. However, Prevete et al. found that *FPR1* acted as a tumor suppressor in human gastric cancer by counteract angiogenesis^24^.

*TNFRSF6B* belongs to the tumor necrosis factor receptor superfamily and acts as inhibiting Fas ligand-induced apoptosis^25^. Upregulated *TNFRSF6B* was identified in several human cancers including colon and lung cancers, and functioned as predictor of tumor invasion^26^. Tseng and colleagues found that overexpression of *TNFRSF6B* was also associated with the progression of chronic kidney disease^27^. Zekri et al. studied the differentially expressed genes in metastasis advanced Egyptian BCa and found that *TNFRSF6B* was downregulated in BCa samples, which was contrary to our findings^28^. This study, however, did not explore the molecular mechanism of *TNFRSF6B* in depth.

Together, we identified five PIRMGs, including *FPR1*, *CIITA*, *KLRC1*, *TNFRSF6B*, and *WFIKKN1*, which might play a vital role in tumorigenesis of BCa and serve as potential targets of immunotherapy. Besides, we developed a prognosis signature based on a series of analyses, which had good prognosis prediction ability in BCa. Ultimately, a stable prognostic predictive nomogram of signature, age, and stage, was developed to predict the 1-, 3- and 5-year survival probability of patient with BCa. Further researches are needed to verify the clinical effectiveness of this nomogram.

## Methods

**Figure 8:**
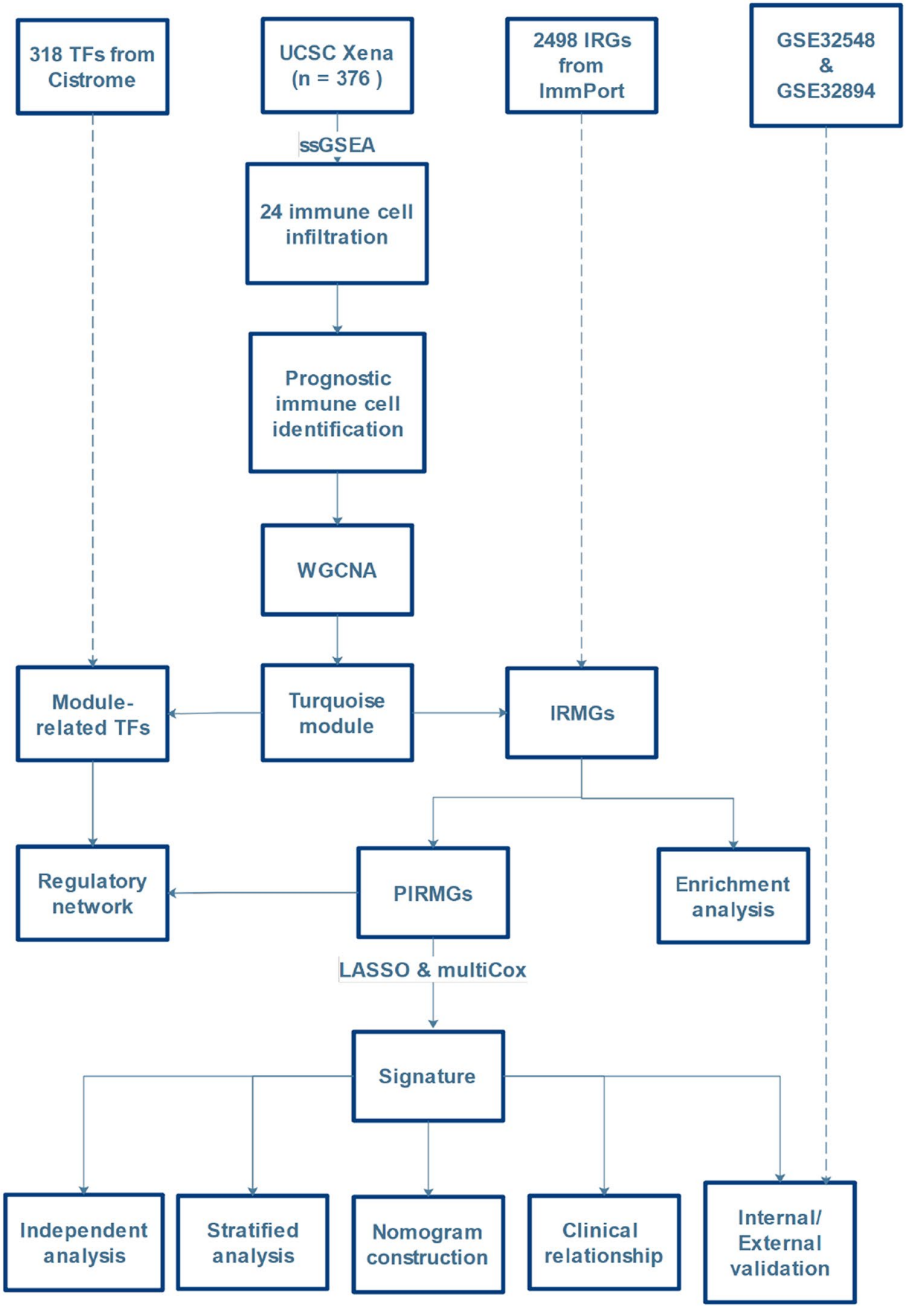
Work flow of this study. *TF* transcription factors, *IRG* immune-related gene, *ssGSEA* single sample gene set enrichment analysis, *WGCNA* weighted correlation network analysis, *IRMG* immune-related module gene, *PIRMG* prognostic IRMG, *LASSO* Least Absolute Shrinkage and Selector Operation, *multiCox* multivariate Cox.

Figure 8 presents the work flow of our study.

### Data acquisition

We downloaded the transcriptome data (log2 transformed RSEM normalized count) and clinical data of BCa from the TCGA Hub in the UCSC Xena database (https://tcga.xenahubs.net). GSE32548 and GSE32894 were downloaded from GEO database (https://www.ncbi.nlm.nih.gov/gds/). The exclusive criteria of BCa samples were: (1) BCa with follow up < 90 days and, (2) BCa with missing survival data. Finally, 376, 126 and 224 BCa samples in TCGA and GSE32548 and GSE32894 datasets were obtained, respectively. Samples of TCGA dataset were divided into training and testing cohorts. The training cohort was used for signature construction, and the testing cohort and entire TCGA dataset were used for internal validations. Tow GEO datasets were used for external validations. Ethics approval statement is not needed because the BCa samples were obtained from the public databases.

A total of 2498 immune-related genes (IRGs) were obtained from the Immunology Database and Analysis Portal (ImmPort) database (https://www.immport.org/)^29^. 318 transcription factors (TFs) were downloaded from the Cancer database (http://cistrome.org)^30^ for constructing a regulatory network.

### Infiltration levels of 24 immune cells in BCa samples

Firstly, we quantified the infiltration abundances of the 24 immune cells (reported in previous studies) in BCa samples by ssGSEA^31,32^. After the infiltration levels and the survival data of the BCa samples were obtained and merged, univariate Cox regression analysis and Kaplan–Meier survival analysis were performed to screen the shared prognostic immune cells. KM curves were produced to illustrate the results of survival analyses.

Then, with an attempt to further clarify the possible association between the shared prognostic cells and tumor progression, we analyzed the relationship between clinicopathological factors (age, grade, stage and subtypes) and these cells.

### Construction of a weighted co-expression network

The input data files of WGCNA were: (1) transcriptome data of the 376 BCa samples of TCGA dataset and (2) the phenotype matrix which consisted of the survival data and the infiltration levels of the identified prognostic immune cells.

Firstly, the variability of gene expressions across the 376 samples was measured by a robust method called median absolute deviation (MAD). And the top 5000 MAD genes were identified for following analysis. A sample dendrogram with phenotype heatmap was constructed.

Subsequently, we calculated the best soft-thresholding, named β, by Soft Threshold function. A weighted adjacency matrix was then constructed based on the β value. And the adjacency matrix was further transformed into a topological overlapping matrix (TOM). Finally, genes with similar expressions were clustered into one module called co-expression module. p‑values and correlation coefficients were calculated to identify the association between a co-expression module and the phenotype. Finally, the key module with significant correlation with phenotype was identified, and all genes within the module (module genes, MGs) were extracted for next studies.

### Identification of immune-related module genes (IRMGs)

IRMGs were considered as the shared genes of MGs and IRGs. A Venn plot by “VennDiagram” package in R was generated to show the analytic results^33^. To reveal the functional mechanism of the IRMGs, we then performed enrichment analysis by online tool Metascape (https://metascape.org/) which is designed to provide a comprehensive gene list annotation and analysis resource for researchers^34^.

### Identification of prognostic IRMGs (PIRMGs)

The 376 BCa samples of TCGA dataset were randomly divided into training cohort and testing cohort in the ratio of 1:1. Then, we performed univariate Cox regression analysis in the training cohort to screen the IRMGs associated with prognosis of BCa, and IRMGs with p-values lower than 0.05 were considered as PIRMGs. The online tool cBioportal (http://www.cbioportal.org/) was then used to analyze the alterations of the PIRMGs^35^.

Next, we identified TFs in the key module by “VennDiagram” package and analyzed their correlations with PIRMGs by Pearson correlation coefficient. We further selected significant correlations under the cutoff values: correlation score > 0.4 and p-value < 0.001. A regulatory network of the significant correlations was ultimately constructed to further explore the molecular mechanisms of these PIRMGs. Software Cytoscape 3.8.0 was employed to visualize the network^36^.

### Development of a prognostic signature

A prognostic signature was developed by Least Absolute Shrinkage and Selector Operation (LASSO) analysis and multivariate Cox regression analysis. Risk score of each BCa was calculated using the formula: Risk score = ExpGene_1_ × CoefGene_1_ + ExpGene_2_ × CoefGene_2_ + … ExpGene_(n)_ × CoefGene_(n)_. In this equation, “ExpGene” represented gene expression and “CoefGene” was the regression coefficient.

Then, BCa samples in training cohort were divided into high- and low-risk groups depending on the median value of the risk score. KM survival curve by log-rank test and time‐dependent receiver operating characteristic (ROC) curve were produced to assess the prognostic ability and performance of the signature. Further, the stability and reliability of the signature was verified in the internal and external validation group by the same methods.

Furthermore, the independent prognostic ability of signature was assumed by univariate and multivariate Cox regression analysis. Stratified analysis was also employed to assess the performance of signature in the same clinicopathological characteristics.

### Development and evaluation of a nomogram

With an attempt to predict the 1-, 3- and 5-year survival probability of each BCa patient, we constructed a nomogram based on the risk score and independent clinicopathological characteristics. The performance of the nomogram was validated using time‐dependent ROC curves and calibration plots.

Additionally, to further asses the clinical utility of the signature for prognosis, we compared a full model including risk score and clinical variables (age, stage and grade) to a base model including only clinical variables through time-dependent ROC curves. AUC represents the performance of each model at specific time point.

## Supplementary Information

Supplementary Figure 1.

Supplementary Figure 2.

Supplementary Figure 3.

Supplementary Figure 4.

## Data Availability

The data underlying this study are freely available from the TCGA Hub at Xena datasets (https://tcga.xenahubs.net) and the GEO database with accession number of GSE32548 and GSE32894 (http://www.ncbi.nlm.nih.gov/geo/).

## Abbreviations

BCa: Bladder cancer
NMIBC: Non- muscle-invasive bladder cancer
MIBC: Muscle-invasive bladder cancer
ssGSEA: Single sample gene set enrichment analysis
WGCNA: Weighted correlation network analysis
IRGs: Immune-related genes
TFs: Transcription factors
GSEA: Gene set enrichment analysis
MAD: Median absolute deviation
MGs: Module genes
IRMGs: Immune-related module genes
PIRMGs: Prognostic IRMGs
LASSO: Least absolute shrinkage, and selector operation
AUC: Area under curve
ROC: Receiver operating characteristic
MHC: Major histocompatibility complex

## References

[1] Saginala K (2020). Epidemiology of bladder cancer. Med. Sci. (Basel).

[2] Seidl C (2020). Targets for therapy of bladder cancer. Semin. Nucl. Med..

[3] Stenzl A (2011). Treatment of muscle-invasive and metastatic bladder cancer: Update of the EAU guidelines. Eur. Urol..

[4] Soukup V (2017). Prognostic performance and reproducibility of the 1973 and 2004/2016 World Health Organization Grading classification systems in non-muscle-invasive bladder cancer: A European Association of Urology non-muscle invasive bladder cancer guidelines panel systematic review. Eur. Urol..

[5] Kouznetsova VL, Kim E, Romm EL, Zhu A, Tsigelny IF (2019). Recognition of early and late stages of bladder cancer using metabolites and machine learning. Metabolomics.

[6] Smith CC (2019). Endogenous retroviral signatures predict immunotherapy response in clear cell renal cell carcinoma. J. Clin. Investig..

[7] Lu X (2017). Effective combinatorial immunotherapy for castration-resistant prostate cancer. Nature.

[8] Li F, Guo H, Wang Y, Liu B, Zhou H (2020). Profiles of tumor-infiltrating immune cells and prognostic genes associated with the microenvironment of bladder cancer. Int. Immunopharmacol..

[9] Jiang W, Zhu D, Wang C, Zhu Y (2020). An immune relevant signature for predicting prognoses and immunotherapeutic responses in patients with muscle-invasive bladder cancer (MIBC). Cancer Med..

[10] Yamamoto K (1994). Stat4, a novel gamma interferon activation site-binding protein expressed in early myeloid differentiation. Mol. Cell. Biol..

[11] Zhang N, Bevan MJ (2011). CD8(+) T cells: Foot soldiers of the immune system. Immunity.

[12] Knutson KL, Disis ML (2005). Tumor antigen-specific T helper cells in cancer immunity and immunotherapy. Cancer Immunol. Immunother..

[13] Szláma G, Kondás K, Trexler M, Patthy L (2010). WFIKKN1 and WFIKKN2 bind growth factors TGFβ1, BMP2 and BMP4 but do not inhibit their signalling activity. FEBS J..

[14] Gornalusse GG (2017). HLA-E-expressing pluripotent stem cells escape allogeneic responses and lysis by NK cells. Nat. Biotechnol..

[15] Kamiya T, Seow SV, Wong D, Robinson M, Campana D (2019). Blocking expression of inhibitory receptor NKG2A overcomes tumor resistance to NK cells. J. Clin. Investig..

[16] Chen Y (2020). CD8+ T cells form the predominant subset of NKG2A+ cells in human lung cancer. Front. Immunol..

[17] Mortara L (2006). CIITA-induced MHC class II expression in mammary adenocarcinoma leads to a Th1 polarization of the tumor microenvironment, tumor rejection, and specific antitumor memory. Clin. Cancer Res..

[18] Accolla RS, Ramia E, Tedeschi A, Forlani G (2019). CIITA-driven MHC class II expressing tumor cells as antigen presenting cell performers: Toward the construction of an optimal anti-tumor vaccine. Front. Immunol..

[19] Lee YS, Kim SH, Cho JA, Kim CW (2011). Introduction of the CIITA gene into tumor cells produces exosomes with enhanced anti-tumor effects. Exp. Mol. Med..

[20] Jiang X, Lei T, Zhang M (2018). Expression and functions of formyl peptide receptor 1 in drug-resistant bladder cancer. Technol. Cancer Res. Treat..

[21] Minopoli M (2019). Targeting the Formyl Peptide Receptor type 1 to prevent the adhesion of ovarian cancer cells onto mesothelium and subsequent invasion. J. Exp. Clin. Cancer Res..

[22] Cao G, Zhang Z (2018). FPR1 mediates the tumorigenicity of human cervical cancer cells. Cancer Manag. Res..

[23] Morris S (2018). Whole blood FPR1 mRNA expression predicts both non-small cell and small cell lung cancer. Int. J. Cancer.

[24] Prevete N (2015). The formyl peptide receptor 1 exerts a tumor suppressor function in human gastric cancer by inhibiting angiogenesis. Oncogene.

[25] Pitti RM (1998). Genomic amplification of a decoy receptor for Fas ligand in lung and colon cancer. Nature.

[26] Migone T-S (2002). TL1A is a TNF-like ligand for DR3 and TR6/DcR3 and functions as a T cell costimulator. Immunity.

[27] Tseng W-C, Yang W-C, Yang A-H, Hsieh S-L, Tarng D-C (2013). Expression of TNFRSF6B in kidneys is a novel predictor for progression of chronic kidney disease. Mod. Pathol..

[28] Zekri A (2015). Differentially expressed genes in metastatic advanced Egyptian bladder cancer. Asian Pac. J. Cancer Prev..

[29] Bhattacharya S (2018). ImmPort, toward repurposing of open access immunological assay data for translational and clinical research. Sci. Data.

[30] Liu T (2011). Cistrome: An integrative platform for transcriptional regulation studies. Genome Biol..

[31] Chen X (2020). Bioinformatics analysis finds immune gene markers related to the prognosis of bladder cancer. Front. Genet..

[32] Bindea G (2013). Spatiotemporal dynamics of intratumoral immune cells reveal the immune landscape in human cancer. Immunity.

[33] Chen H, Boutros PC (2011). VennDiagram: A package for the generation of highly-customizable Venn and Euler diagrams in R. BMC Bioinform..

[34] Zhou Y (2019). Metascape provides a biologist-oriented resource for the analysis of systems-level datasets. Nat. Commun..

[35] Gao J (2013). Integrative analysis of complex cancer genomics and clinical profiles using the cBioPortal. Sci. Signal.

[36] Shannon P (2003). Cytoscape: A software environment for integrated models of biomolecular interaction networks. Genome Res..

