# Supplementary material for: An immune cell infiltration-related gene signature predicts prognosis for bladder cancer
Source: Sci Rep. 2021 Aug 17;11:16679. doi: 10.1038/s41598-021-96373-w (PMC8370985; doi:10.1038/s41598-021-96373-w)
Supplement: Supplementary file 2 — Supplementary Figure 2. [file 41598_2021_96373_MOESM2_ESM.pdf]

An Immune Cell Infiltration-Related Gene Signature Predicts Prognosis for Bladder Cancer

Hualin Chen [1], Yang Pan [1], Xiaoxiang Jin [1], Gang Chen [1*]

1 Department of Urology, The First Affiliated Hospital of Chongqing Medical University, Chongqing, China

*** Correspondence:**
Gang Chen
chengang2308@163.com

+86-13668039053

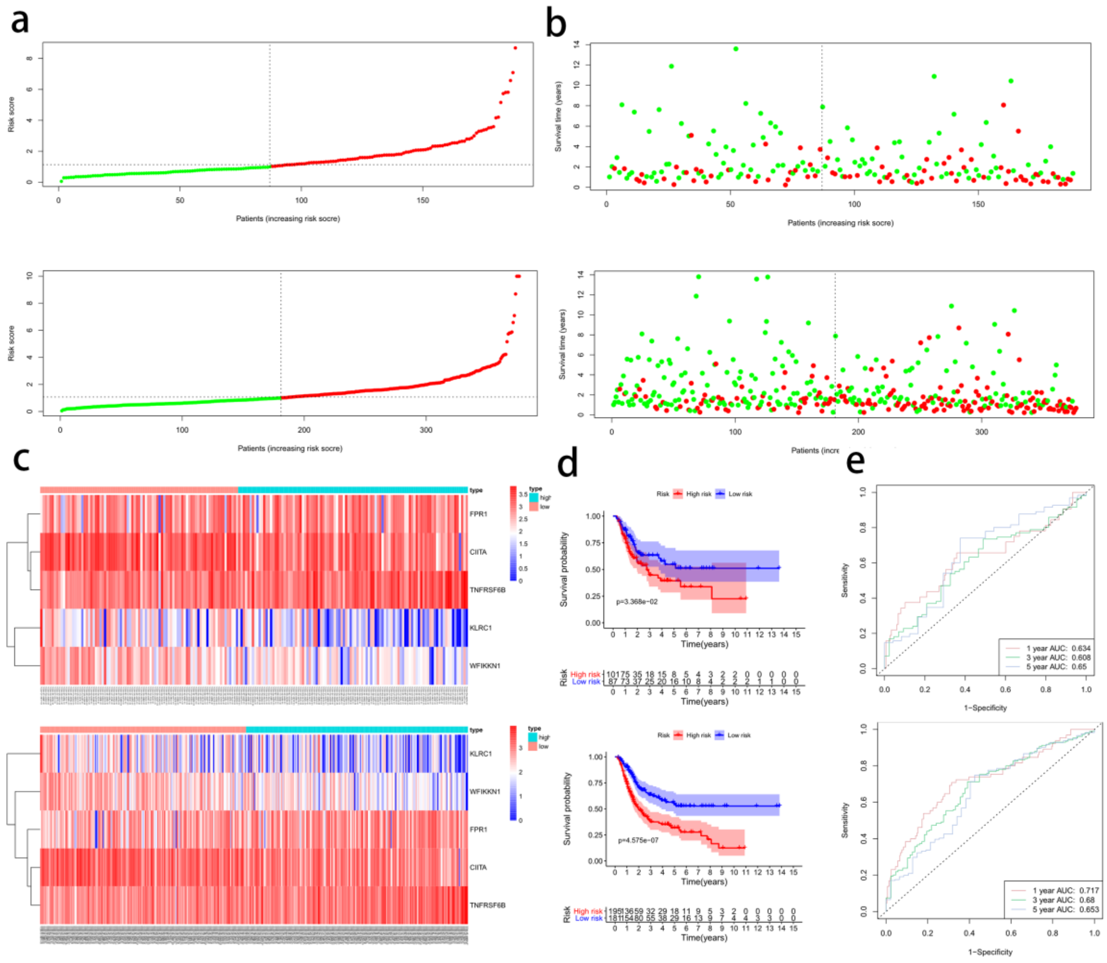

Supplementary Figure 2
Evaluation of the prognosis prediction power of the five PIRMGs signature in the testing and entire cohorts. (**a**) Distribution of risk score. (**b**) Survival status of BCa patients. (**c**) The expression profiles of the five PIRMGs. (**d**) KM survival analysis of the high- and low-risk groups. (**e**) Time-dependent ROC curve of the signature. In each

panel, the upper and lower images correspond to the training and entire cohorts, respectively.
