# Supplementary material for: An immune cell infiltration-related gene signature predicts prognosis for bladder cancer
Source: Sci Rep. 2021 Aug 17;11:16679. doi: 10.1038/s41598-021-96373-w (PMC8370985; doi:10.1038/s41598-021-96373-w)
Supplement: Supplementary file 3 — Supplementary Figure 3. [file 41598_2021_96373_MOESM3_ESM.pdf]

# An Immune Cell Infiltration-Related Gene Signature Predicts Prognosis for Bladder Cancer

**Hualin Chen [1], Yang Pan [1], Xiaoxiang Jin [1], Gang Chen [1*]**

1 Department of Urology, The First Affiliated Hospital of Chongqing Medical University, Chongqing, China

*** Correspondence:**
Gang Chen
chengang2308@163.com

+86-13668039053

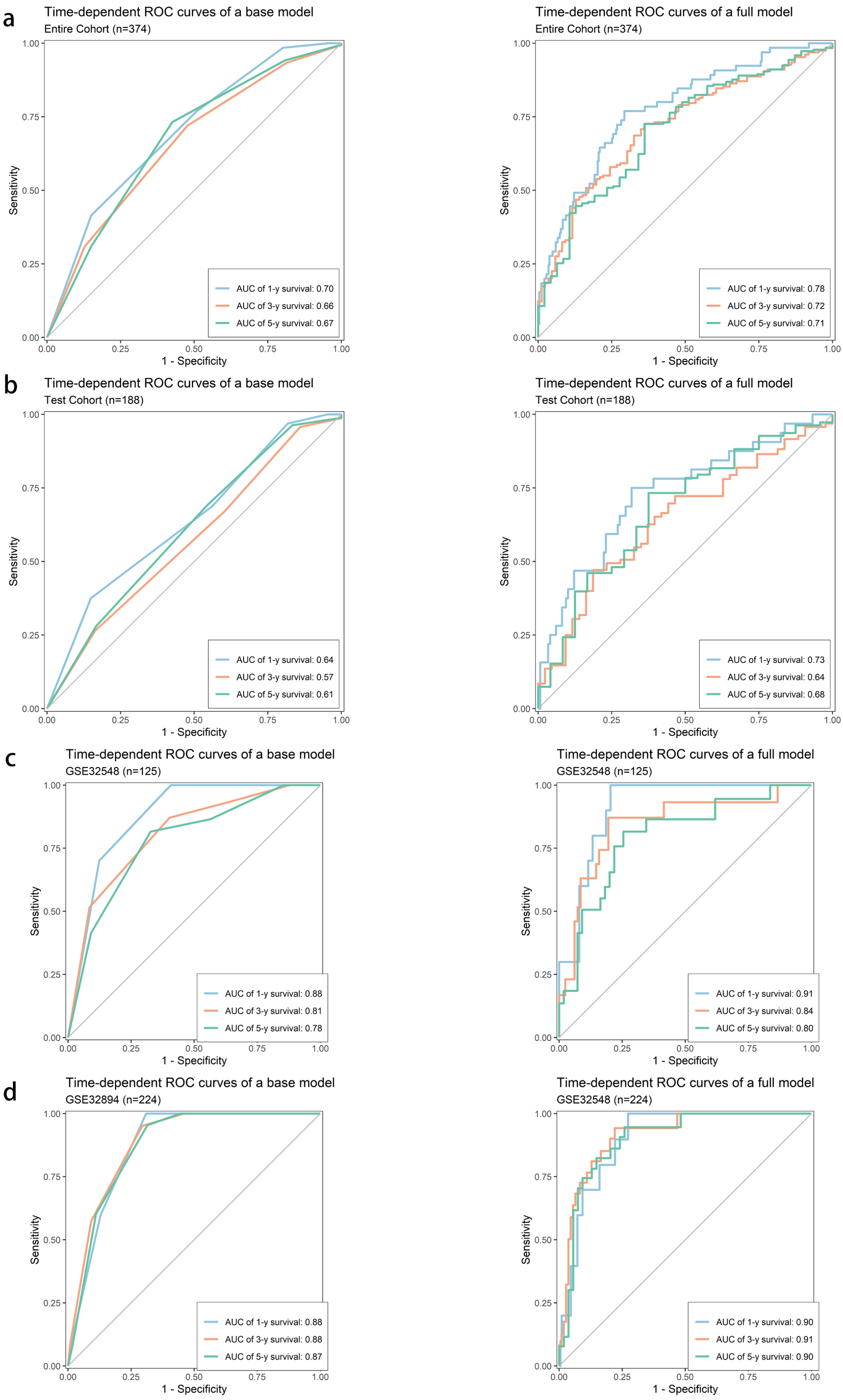


Supplementary Figure 3

Time-dependent ROC curves of a full model (left panel) and a base model (right panel) in the prediction of prognosis at 1-, 3-, and 5-year time points of (a) entire cohort, (b) test cohort, (c) GSE32548 and (d) GSE32894. The full model composed of signature and clinical variables including age, stage and grade. While the base model only composed of clinical variables.
