# Supplementary material for: An immune cell infiltration-related gene signature predicts prognosis for bladder cancer
Source: Sci Rep. 2021 Aug 17;11:16679. doi: 10.1038/s41598-021-96373-w (PMC8370985; doi:10.1038/s41598-021-96373-w)
Supplement: Supplementary file 4 — Supplementary Figure 4. [file 41598_2021_96373_MOESM4_ESM.pdf]

An Immune Cell Infiltration-Related Gene Signature Predicts Prognosis for Bladder Cancer

Hualin Chen [1], Yang Pan [1], Xiaoxiang Jin [1], Gang Chen [1*]

1 Department of Urology, The First Affiliated Hospital of Chongqing Medical University, Chongqing, China

*** Correspondence:**
Gang Chen
chengang2308@163.com

+86-13668039053

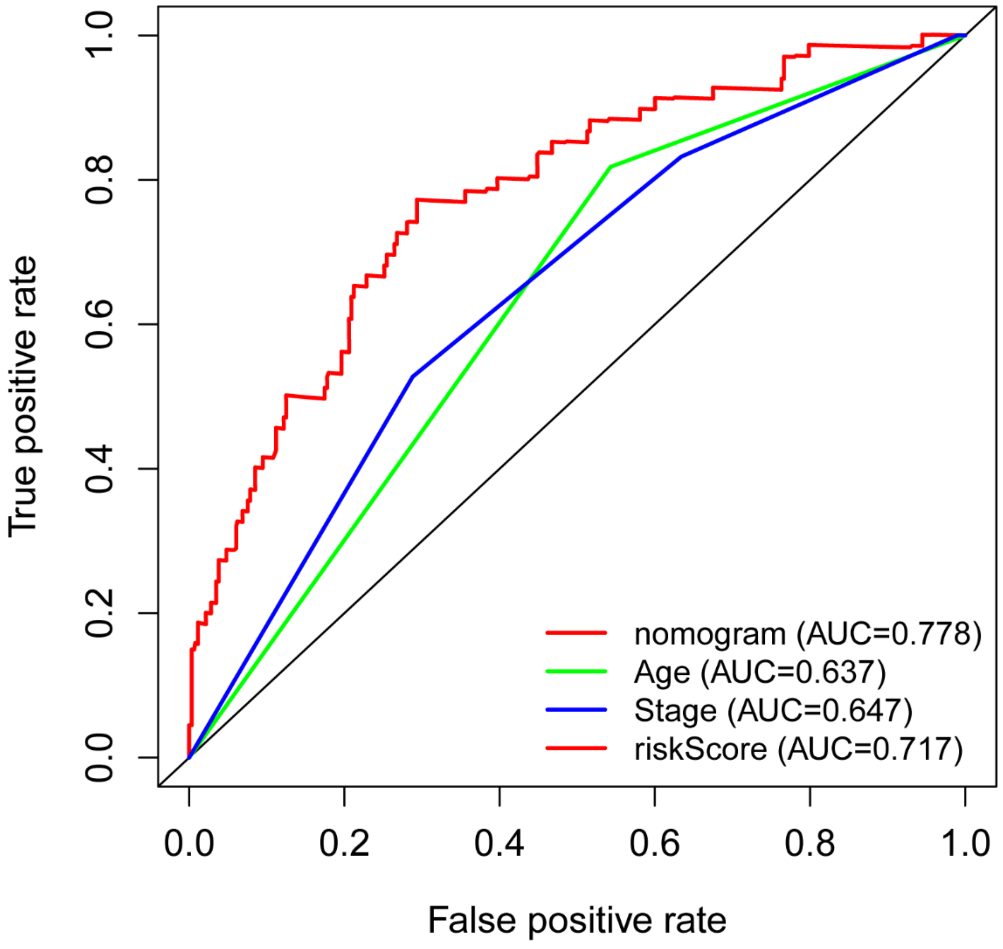

Supplementary Figure 4. Evaluation the performance of the signature and nomogram by time-dependent ROC curves.
